# Radiation induces iatrogenic immunosuppression by indirectly affecting hematopoiesis in bone marrow

**DOI:** 10.18632/oncotarget.27564

**Published:** 2020-05-12

**Authors:** Vaishali Kapoor, Andrea Collins, Kaylan Griffith, Subhajit Ghosh, Nathan Wong, Xiaowei Wang, Grant A. Challen, Joseph Krambs, Dan Link, Dennis E. Hallahan, Dinesh Thotala

**Affiliations:** ^1^Department of Radiation Oncology, St. Louis School of Medicine, Washington University, St. Louis, Missouri, USA; ^2^Siteman Cancer Center, St. Louis School of Medicine, Washington University, St. Louis, Missouri, USA; ^3^Department of Medicine, St. Louis School of Medicine, Washington University, St. Louis, Missouri, USA; ^4^Department of Pathology & Immunology, St. Louis School of Medicine, Washington University, St. Louis, Missouri, USA; ^5^Medical Scientist Training Program, St. Louis School of Medicine, Washington University, St. Louis, Missouri, USA

**Keywords:** radiation, lymphopenia, mass cytometry RNA sequencing

## Abstract

The immune system plays a vital role in cancer therapy, especially with the advent of immunotherapy. Radiation therapy induces iatrogenic immunosuppression referred to as radiation-induced lymphopenia (RIL). RIL correlates with significant decreases in the overall survival of cancer patients. Although the etiology and severity of lymphopenia are known, the mechanism(s) of RIL are largely unknown. We found that irradiation not only had direct effects on circulating lymphocytes but also had indirect effects on the spleen, thymus, and bone marrow. We found that irradiated cells traffic to the bone marrow and bring about the reduction of hematopoietic stem cells (HSC) and progenitor cells. Using mass cytometry analysis (CyTOF) of the bone marrow, we found reduced expression of CD11a, which is required for T cell proliferation and maturation. RNA Sequencing and gene set enrichment analysis of the bone marrow cells following irradiation showed down-regulation of genes involved in hematopoiesis. Identification of CD11a and hematopoietic genes involved in iatrogenic immune suppression can help identify mechanisms of RIL.

## INTRODUCTION

The immune system plays an important role in keeping us healthy from pathogens and foreign bodies including cancer cells. Immune system plays an important role in cancer therapy especially with the advent of immunotherapy [1]. Radiation causes iatrogenic immunosuppression referred to as Radiation-induced lymphopenia (RIL), which correlates with treatment outcomes and survival of cancer patients [2–4]. RIL is associated with poor outcome for many cancers, including lung, colon, pancreas, and breast [2–4]. An improved understanding of the biological mechanisms of the persistence of RIL is needed.

Previous studies have implicated the direct effects of radiation on lymphocyte depletion [5, 6]. Direct damage to the T-cells from radiation will reduce the number of T-cells in circulation which should be repopulated in 80–90 days. However, RIL persists six months to a year after irradiation [3, 7] which is far beyond the T-cell turnover time suggesting an indirect mechanism may be involved in immune suppression.

Previously we found that autologous transplantation of hematopoietic stem cells after irradiation rescued mice from RIL [8]. A similar approach was successful in overcoming chemotherapy-induced lymphopenia. Autologous hematopoietic stem cell reinfusion to lymphopenic patients with either advanced myeloma [9] or metastatic breast cancer [10] after high dose chemotherapy achieved the recovery of lymphocytes.

Previous studies have focused on the direct effect of radiation on T-cells [5, 6]. Our central hypothesis is that long-term RIL involves not only T-cells but also indirect effects on hematopoietic stem cells. To study these phenomena, we utilized CyTOF and RNA sequencing to identify the changes in the bone marrow after irradiation. We also analyzed the T and B cells from blood-forming organs that include blood, spleen, thymus, and bone marrow.

We found that irradiation affects the T-cells and B-cells in the spleen, thymus, and bone marrow. We also found cellular and transcriptome changes in the bone marrow compartment of mice in response to thoracic IR. This study identified depletion the of CD11a expression in the HSC and progenitor cells in the bone marrow and downregulation of expression of hematopoiesis genes in the bone marrow stem cells.

## RESULTS

### Irradiation depletes cells in blood, spleen, and thymus

**Figure 1:**
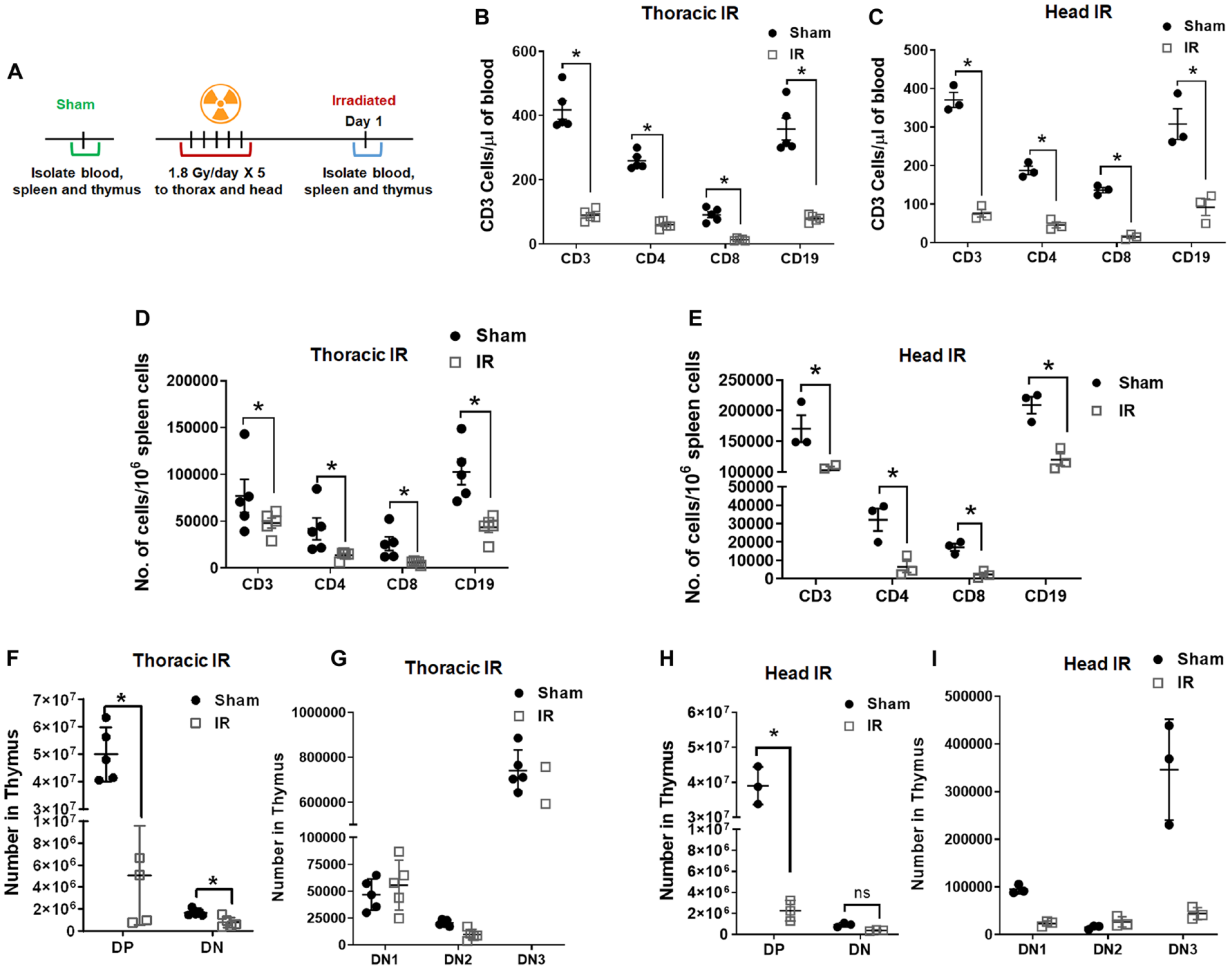
Radiation depletes cells in blood, spleen, and thymus. Schematic representation of the treatment plan for mice (**A**). The mouse thorax or head was irradiated with 1.8 Gy for 5 days consequently. The blood, spleen and thymus from the mice were analyzed 1 day post irradiation, untreated mice were used as controls. Irradiation depletes CD3, CD4, CD8 and CD19 in the blood (**B**, **C**) and spleen (**D**, **E**). Irradiation depletes double positive (DP) and double negative (DN) populations (**F**, **G**) along with DN1, DN2, and DN3 populations (**H**, **I**) in thymus. SD are from at least three treatments.

To determine the effect of radiation on the lymphoid organs, we analyzed the blood, spleen, and thymus after irradiation. The C57BL/6 mice were irradiated with 5 doses of 1.8 Gy to the thorax or head, and sham-irradiated mice served as controls (Figure 1A and Supplementary Figure 1). The blood and spleen were analyzed for CD3+, CD4+, CD8+ T cells, and CD19+ B cells using flow cytometry 24 h post final irradiation. T cells (for cell-mediated, cytotoxic adaptive immunity), and B cells (for humoral, antibody-driven adaptive immunity) are the main types of cells found in the blood. After irradiation to the thorax, we found a significant reduction in the circulating CD3 (4.18E+02 vs. 9.00E+01; *P* ≤ 0.0001), CD4 (2.59E+02 vs. 6.06E+01; *P* ≤ 0.0001), CD8 (9.14E+01 vs. 1.36E+01; *P* = 0.011) and CD19 (3.58E+02 vs. 7.99E+01; *P* ≤ 0.0001) cells in the blood (Figure 1B). After irradiation to the head, we found a significant reduction in the circulating CD3 (3.71E+02 vs. 7.64E+01; P ≤ 0.0001), CD4 (1.88E+02 vs. 4.63E+01; *P* ≤ 0.0003), CD8 (1.37E+02 vs. 1.51E+01; *P* = 0.0012) and CD19 (3.08E+02 vs. 9.23E+01; *P* ≤ 0.0001) cells in the blood (Figure 1C).

The spleen is a secondary lymphoid organ that plays an important role in clearing the damaged cells and plays an important role in the adaptive immune response. After irradiation to thorax, we found a significant reduction in CD3 (7.71E+04 vs. 4.81E+04; *P* ≤ 0.001), CD4 (4.19E+04 vs. 1.38E+04; *P* ≤ 0.001), CD8 (2.60E+04 vs. 6.11E+03; *P* ≤ 0.001) and CD19 (1.03E+05 vs. 4.35E+04; *P* ≤ 0.001) cells in the spleen (Figure 1D). After irradiation to the head, we found a significant reduction in CD3 (1.71E+05 vs. 1.03E+05; *P* ≤ 0.001), CD4 (3.21E+04 vs. 6.56E+03; *P* ≤ 0.002), CD8 (1.72E+04 vs. 2.36E+03; *P* ≤ 0.02) and CD19 (2.09E+05 vs. 1.20E+05; *P* ≤ 0.006) cells in the spleen (Figure 1E). Analysis of the spleen following thoracic irradiation (Supplementary Figure 2A) showed a significant reduction in the size (Supplementary Figure 2B) and weight (88.9 mg vs. 32.3 mg; *P* ≤ 0.0001; Supplementary Figure 2C).

To study the effects of radiation on thymus, we irradiated the mouse thorax and head (1.8Gy ×5) and analyzed the thymus of the mice. T cell progenitors evolve into thymocytes in the Thymus. The T cell development in the thymus takes place in three broad phases that are controlled by two developmental checkpoints. The phases are distinguished based on the CD4/CD8 expression status. The earliest thymocytes are double negative or DN phase (DN1, DN2, and DN3) where the thymocytes express neither CD4 nor CD8. As the thymocytes mature, they express both CD4 and CD8 called the double positive or DP phase. The thymocytes then undergo thymic selection to commit to either the CD4 or CD8 lineage referred to as single positive or the SP phase [11]. After irradiation to the thorax and analysis of the thymus, we found a significant reduction in DP (4.99E+07 vs. 5.06E+06 *P* ≤ 0.001) and DN (1.7E+06 vs. 7.7E+05; *P* ≤ 0.001) cell populations (Figure 1F). In our stepwise analysis of the thymus, we found that all populations of DN1 (4.68E+04 vs. 5.56E+04 *P* = 0.998), DN2 (2.06E+04 vs. 9.61E+03 *P* = 0.997), and DN3 (7.42E+05 vs. 4.42E+05 *P* = 0003), also reduced after thoracic irradiation (Figure 1G). We also found a significant reduction in the size (Supplementary Figure 2D) and weight (Supplementary Figure 2E) of the thymus (89.5 mg vs. 33.9 mg; *P* ≤ 0.0003) after thoracic irradiation. Similarly analysis of the thymus after radiation to the head also, showed significant reduction in DP (3.90E+07 vs. 2.26E+06 *P* ≤ 0.001) and DN (9.16E+05 vs. 3.83E+05; *P* = 0.966) cell populations (Figure 1H). In our stepwise analysis of the thymus, we found that all populations of DN1 (9.50E+04 vs. 2.32E+04 *P* = 0.193), DN2 (1.55E+04 vs. 2.66E+04 *P* = 0.986), and DN3 (3.46E+05 vs. 4.43E+04 *P* ≤ 0.0001), also reduced after irradiation (Figure 1I). These results indicate that IR has direct and indirect effects on T and B cells in lymphoid organs in addition to circulating in blood.

### Irradiation depletes cells in the bone marrow

**Figure 2:**
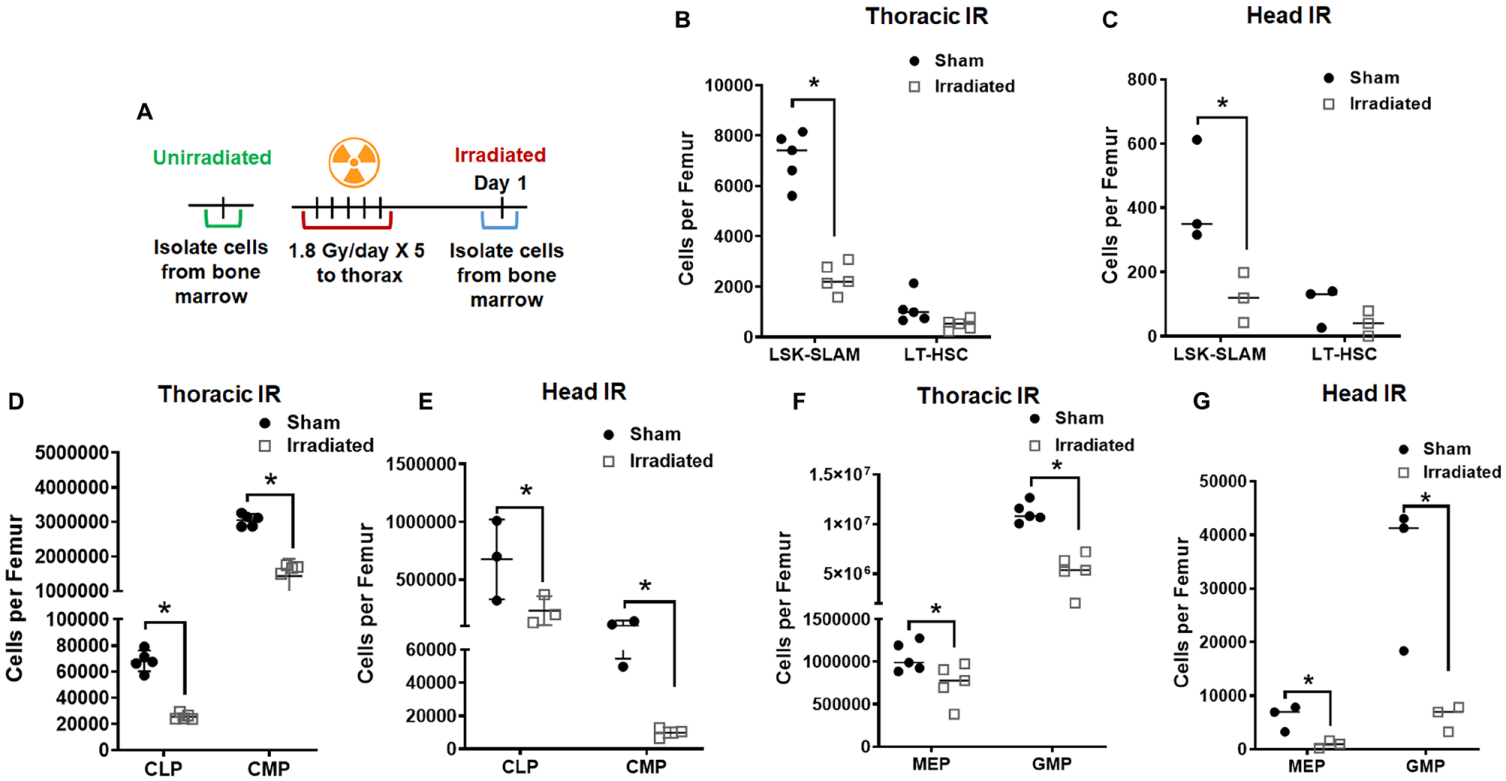
Radiation depletes hematopoietic stem cells and progenitor cells in the bone marrow. Schematic representation of the treatment plan for mice (**A**). The mouse thorax or head were irradiated with 1.8 Gy for 5 consecutive days. The cells from the bone marrow were analyzed using flow cytometry 24 h post final irradiation. Irradiation depleted the LT-HSC and LSK-SLAM cells after thoracic irradiation (**B**) and head irradiation (**C**) total cells when compared to sham mice. Irradiation also depleted both common lymphoid progenitor cells (CLP) and common myeloid progenitor cells (CMP) after thoracic irradiation (**D**) and head irradiation (**E**). Irradiation depleted the megakaryocyte erythroid progenitor (MEP) and granulocyte-monocyte progenitor (GMP) after thoracic irradiation (**F**) and head irradiation (**G**). SD from at least three treatments.

Earlier, we found that long-term lymphopenia is mainly caused by the depletion of hematopoietic stem cells [8]. To determine the indirect effect of radiation on bone marrow, we analyzed Lineage−/lowSca-1+c-Kit+ (LSK) signaling lymphocyte activation molecules (SLAM), long-term hematopoietic stem cells (LT-HSC), common lymphoid progenitor (CLP), common myeloid progenitor (CMP), megakaryocyte erythroid progenitor (MEP) and granulocyte-monocyte progenitor (GMP) cells in bone marrow after irradiation. The C57BL/6 mice were irradiated with 5 doses of 1.8 Gy to the thorax, head, and sham-irradiated mice served as controls (Figure 2A). The mice were irradiated to the thorax or the head and the bone marrow was not directly irradiated.

We found reduction in hematopoietic stem cells LSK-SLAM in thorax irradiated (7.13E+03 vs. 2.36E+03; *P* ≤ 0.0001), and head irradiated (4.26E+02 vs. 1.20E+02; *P* ≤ 0.0001) mice. We also found reduction in LT-HSC in thorax irradiated (1.13E+03 vs. 4.97E+02; *P* ≤ 0.0001) and head irradiated (9.90E+01 vs. 3.97E+01; *P* ≤ 0.0001) mice (Figure 2B and 2C).

We found reduction in lymphoid progenitors CLP in thorax irradiated (6.82E+04 vs. 2.55E+04; *P* = 0.035), and head irradiated (6.76E+05 vs. 2.34E+05; *P* ≤ 0.0001) mice. We also found reduction in myeloid progenitors CMP in thorax irradiated (3.05E+06 vs. 1.44E+06; *P* ≤ 0.0001) and head irradiated (1.01E+05 vs. 9.82E+03; *P* = 0.035) mice (Figure 2D and 2E).

We found reduction in late hematopoietic progenitors MEP in thorax irradiated (1.05E+06 vs. 7.48E+05; *P* = 0.0119), and head irradiated (5.99E+03 vs. 9.15E+02; *P* ≤ 0.0001) mice. We also found reduction in GMP in thorax irradiated (1.12E+07 vs. 5.24E+06; *P* = 0.648) and head irradiated (3.42E+04 vs. 5.99E+03; *P* = 0.0025) mice (Figure 2F and 2G). These results indicate that there is an indirect mechanism of depletion of CLP and CMP in the bone marrow following thoracic and head IR.

### *Ex-vivo* irradiated PBCs traffic to the bone marrow

**Figure 3:**
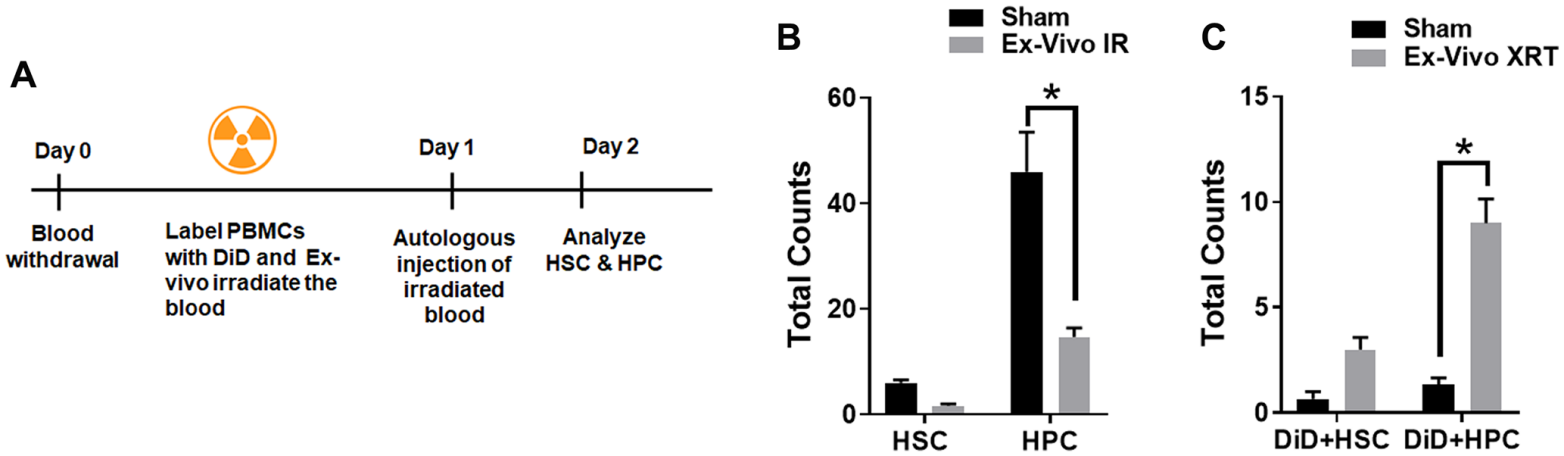
*Ex-vivo* irradiated PBCs traffic to the bone marrow. Schematic representation of the treatment plan for mice (**A**). 200 μl of the whole blood was irradiated *ex vivo* (mice were not irradiated), and the cells were labeled with DiD and reinfused autologously into unirradiated mice. (**B**). *Ex-vivo* irradiated blood induces reduction of hematopoietic stem cells and progenitor cells in mouse bone marrow. (**C**). DiD-labeled hematopoietic stem cells increase in the bone marrow following *ex-vivo* irradiation of blood. SD from at least three treatments.

To determine if irradiated peripheral blood cells traffic to the bone marrow, we isolated peripheral blood cells (PBC) from 200μl of mouse blood, labeled them with a fluorescent membrane dye, DiD and irradiated with sham or 6 Gy irradiation. The PBCs stained with DiD were injected autologously to the mice (Figure 3A). The bone marrow cells were analyzed for hematopoietic stem cells (Lin-Sca1+ckit+CD34-, HSCs) and the hematopoietic progenitor cells (Lin-Sca1+ckit+CD34+, HPCs) at 24 h post autologous injection (Figure 3A). We observed a decrease in both HSC (6 vs. 1.6 cells; ns = 0.7015) and the HPC (46 vs. 14.6 cells; *P* = 0.0009) in the mice injected with *ex-vivo* irradiated peripheral blood mononuclear cells (PBMCs) when compared to sham-irradiated PBMCs (Figure 3B). Therefore we next studied whether *ex-vivo* irradiated PBMCs and/or EVs traffic to the bone marrow and fuse with HSCs and HPCs. We analyzed the presence of the DiD marker on the HSCs and HPCs. We found an increase in the number of DiD+HSC (3 vs. 0.6; ns *P* = 0.084) and DiD+HPCs (9 vs. 1.3; *P* ≤ 0.0001) in the mice injected with irradiated PBMCs compared to sham controls (Figure 3C). The presence of DiD within HSCs and HPCs indicates that *ex vivo* treated blood cells could be trafficking to the bone marrow and bring about its reduction.

### Irradiation depletes CD11a expressing cells in the bone marrow

**Figure 4:**
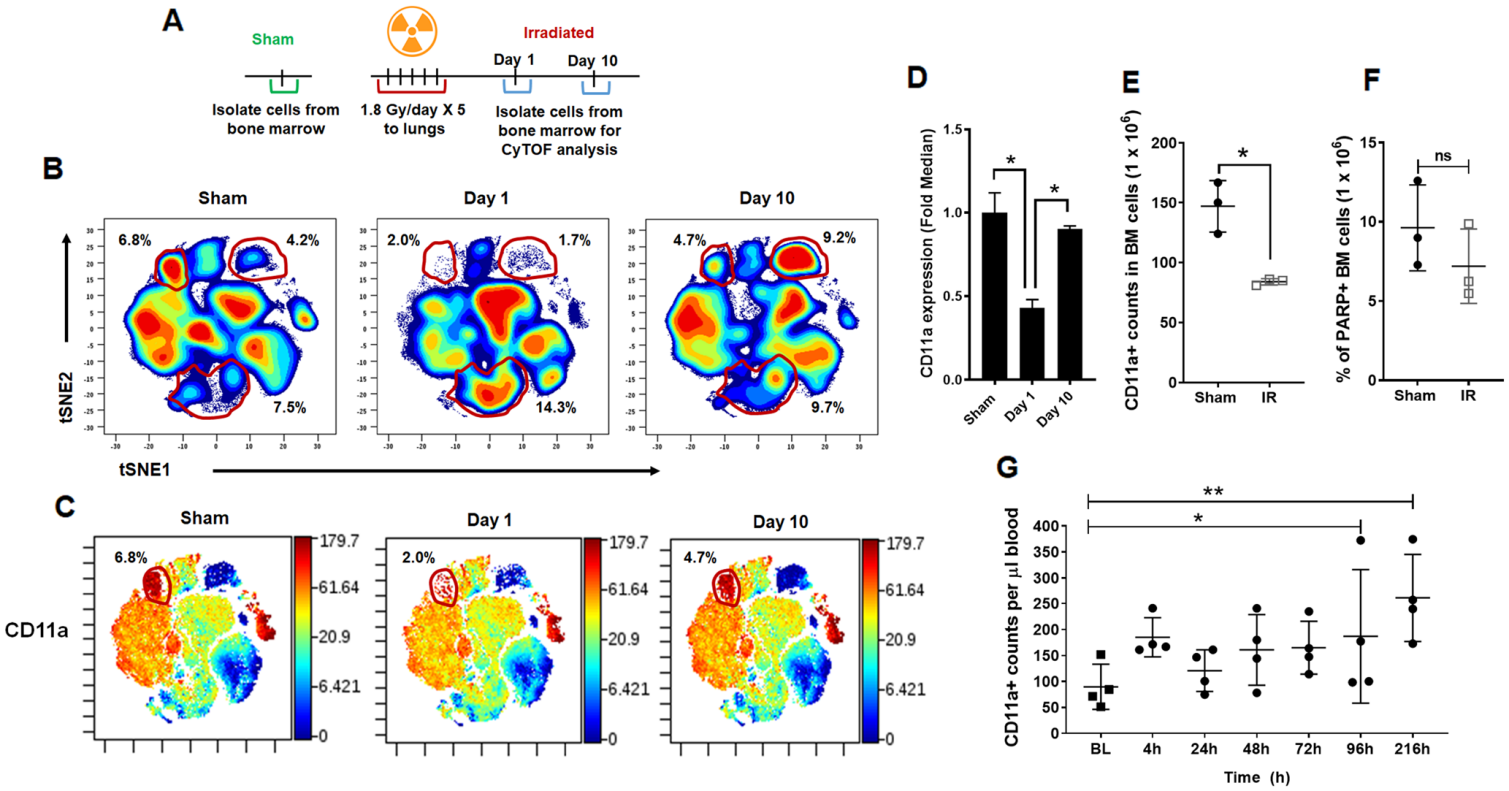
CyTOF analysis of bone marrow cells following irradiation. (**A**) Schematic representation of the treatment of mice prior to mass cytometry analysis (CyTOF) of the bone marrow compartment. Mouse thorax was irradiated, and the cells from femurs of mice were harvested day 1 and day 10 after irradiation, untreated mice were used as controls. (**B**) Representative density plots of lineage negative bone marrow cells in the tSNE1/tSNE2 fields following CyTOF acquisition. (**C**) Lineage-negative bone marrow cells in the tSNE1/tSNE2 fields displaying the median expression of CD11a. Color scale indicates the intensity of expression of CD11a. Minimum (min) and maximum (max) correspond to the 2nd and the 98th percentile values for each indicated marker, respectively. (**D**) Bar graph indicating the fold change in CD11a expression on Day 1 and Day 10 following thoracic IR. (**E**) Flow cytometry analysis of the bone marrow compartment following thoracic IR to validate the results of mass cytometry. The bar graph representing the counts of CD11a+ stem cells in the bone marrow. SD from at least three treatments. (**F**) Flow cytometry analysis of the bone marrow compartment following thoracic IR to evaluate for apoptosis. The bar graph representing the percentage of PARP positive cells in the bone marrow. SD from at least three treatments. (**G**) Irradiation mobilizes the cells expressing CD11a from the bone marrow into the circulation. The mouse thorax were irradiated (1.8 Gy ×5) and the cells expressing CD11a from the circulating blood were analyzed using flow cytometry at the time points indicated.

To study the changes in cell phenotypes in the bone marrow compartment after irradiation, we performed mass cytometry analysis in a Cytometry by Time of Flight (CyTOF). CyTOF uses metal-conjugated antibodies that allow for high-throughput analysis of a large number of parameters on single cells [12]. We developed custom mass cytometry panels that include lymphoid, myeloid, and NK cell lineage, the details are listed in Supplementary Table 1. We irradiated C57BL/6 mice with five fractions of 1.8Gy in thorax region after shielding the rest of the body. We then isolated the bone marrow cells from the femurs of these treated mice on day 1 and day 10 after the last fractionated radiation dose. We also isolated bone marrow cells from untreated mice as sham controls (Figure 4A). Following CyTOF acquisition, the data were analyzed using viSNE clustering (Cytobank). Various population clusters were manually gated in the tSNE1/2 fields, and we identified the differences between treated and untreated mice using the density plots. Figure 4B shows the manual gates on three clusters, which show significant changes on day 1 and day 10, compared to sham. We observed a reduction in cluster 1 at day 1 (2.0%) and day 10 (4.7%) after irradiation when compared to sham (6.8%). We observed a reduction in cluster 2 at day 1 (1.7%) and increase on day 10 (9.2%) after irradiation when compared to sham (4.2%). Cluster 3 was upregulated at day 1 (14.3%) and day 10 (9.7%) compared to sham irradiation (7.5%).

The same viSNE maps were then assessed for the median expression of CD11a (Figure 4C). The bar diagrams show the fold change in the median expression of the indicated markers. CD11a was almost 2-fold downregulated on Day 1 compared to sham mice (Figure 4D). We further validated CD11a in a separate set of mice using flow cytometry. We observed a reduction in CD11a expression in hematopoietic stem cells after irradiation. We found an average of 84 cells/million in irradiated mice when compared to 150/million (*P* = 0.0075) cells in the untreated control (Figure 4E). To determine if the CD11a cells were undergoing apoptosis after IR, we analyzed the bone marrow cells with PARP assay. We found that IR may have induced apoptosis in the bone marrow cells but it was not significantly different from untreated mice (Figure 4F). We then analyzed for cells expressing CD11a being mobilized in circulation after IR (Figure 4G). We found that IR induced the mobilization of CD11a cells from the bone marrow into circulation after IR. At 4h we found that IR led to an initial increase in CD11a cells in circulation (*P* = 0.20), which later stabilized at 24 h, 48 h and 72 h. At 96 h post IR, we found that there was a significant increase of CD11a cells in circulation, which remained significantly elevated even at 216 h (Figure 4G).

### Irradiation causes transcriptome changes in the unirradiated bone marrow compartment

**Figure 5:**
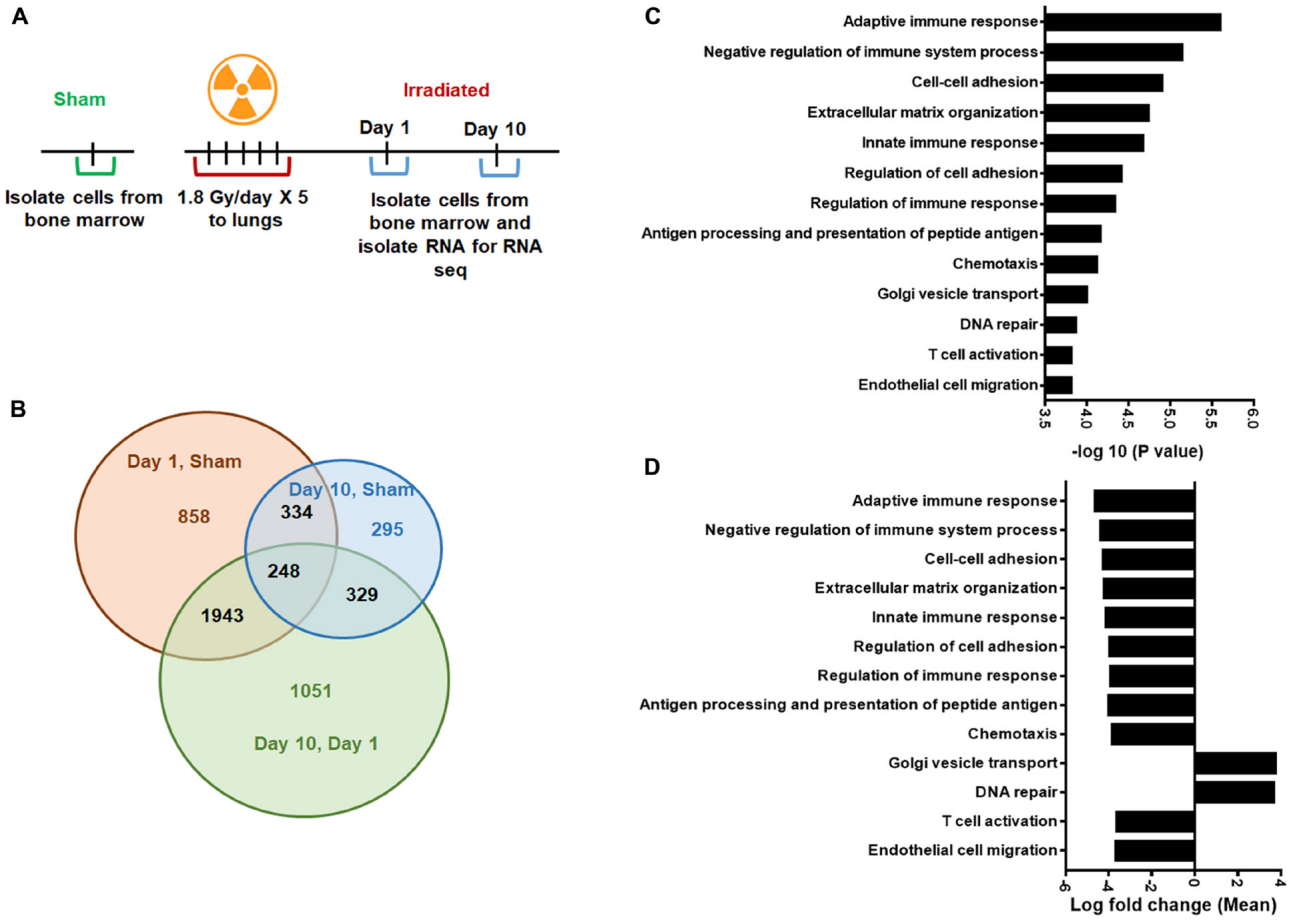
Gene Ontology (GO) analysis to identify differentially regulated genes. (**A**) Schematic representation of the treatment plan. Mouse thorax were irradiated, and the cells from femurs of mice were harvested day 1 and day 10 after irradiation, untreated mice were used as controls. The RNA-sequence analysis was performed on RNA obtained from these bone marrow cells. (**B**) Gene Ontology (GO) analysis was performed to identify differentially regulated mRNAs. Venn diagram showing the number of significantly enriched mRNAs in the indicated groups. (**C**) Bar graph showing –log10 *P*-values of the GO biological processes on day 1 compared to sham mice. (**D**) Bar graph showing the mean log fold change of the GO biological processes shown in panel C. SD from at least three treatments.

To identify the transcriptional changes in the bone marrow compartment after irradiation, we utilized high-throughput RNA sequencing (RNA-Seq). Briefly, the mice were irradiated with 5 doses of 1.8 Gy to the thorax, and sham-irradiated mice served as controls (Figure 5A). The mRNA was isolated from bone marrow cells using the miRVANA kit (Ambion). We performed GO analysis of the RNA-seq data and found that 858 genes changed after day 1 and 295 genes changed after day10 following irradiation when compared with untreated controls. We also observed that the expression of 1051 genes altered in the unirradiated bone marrow after irradiation from day 1 to day 10 (Figure 5B). On comparing day 1 to sham in the GO analysis, we found almost 4-fold upregulation of Golgi vesicle transport and DNA repair genes and downregulation of genes involved in both innate and adaptive immune response, T cell activation, antigen processing, and presentation and other indicated GO processes (Figure 5D). Figure 5C shows the –log 10 *p*-values of the GO biological processes indicated in Figure 5D. The mean log fold change of the GO biological processes at day 10 compared to sham and day 10 compared to day 1 are shown in Supplementary Figure 3. Specifically, on day 10 compared to sham, we observed downregulation of genes that regulate vasculature development, angiogenesis, and Rho protein signaling amongst other signaling pathways.

### Molecular pathways regulated by radiation in bone marrow

**Figure 6:**
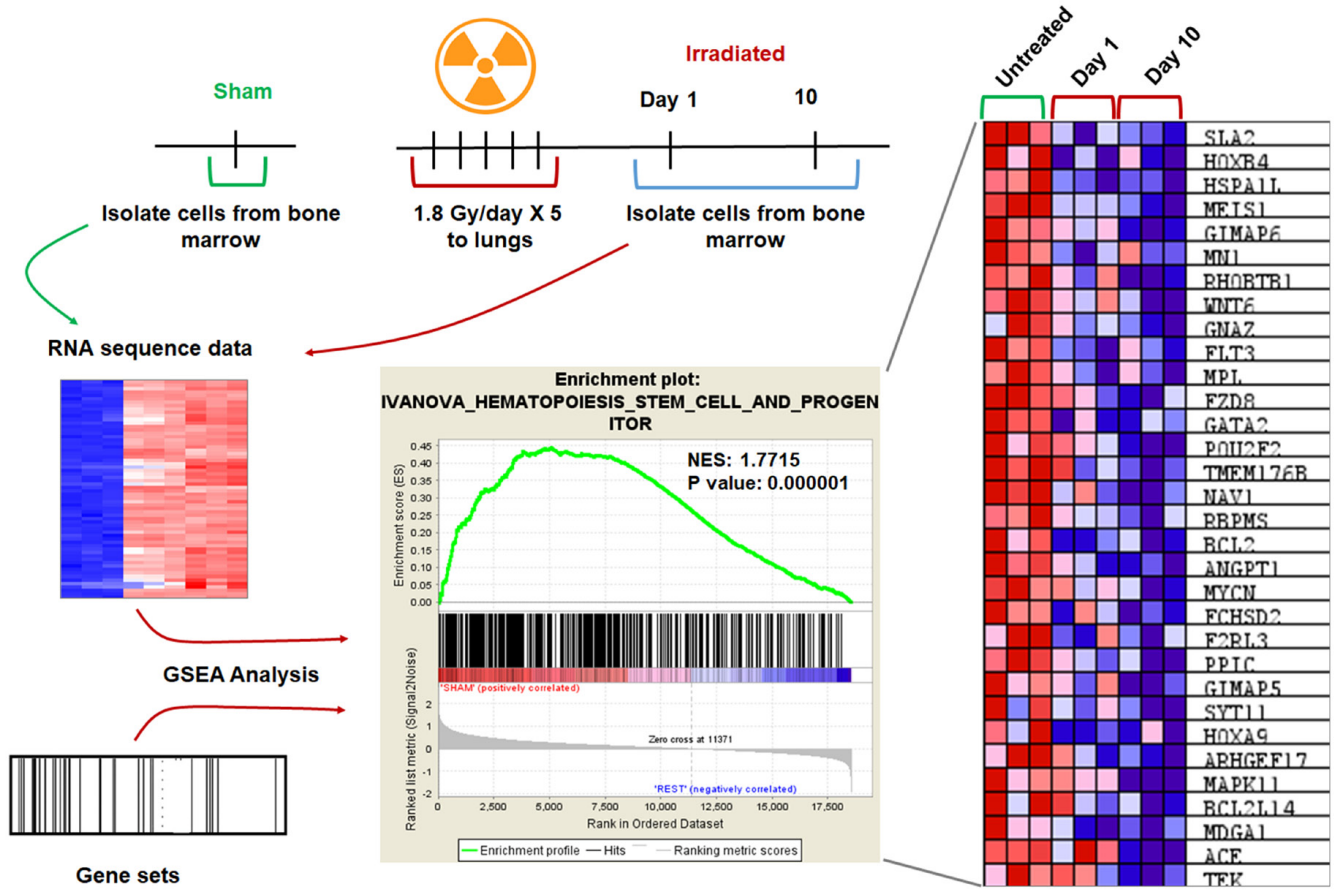
GSEA analysis revealed downregulation of genes involved in hematopoiesis. Mouse lungs were irradiated, and the cells from femurs of mice were harvested day 1 and day 10 after irradiation, untreated mice were used as controls. The RNA-sequence analysis was performed on RNA obtained from these bone marrow cells. Gene Set Enrichment Analysis (GSEA) analysis was performed using RNA-seq data and gene sets for the mouse. Enrichment plot shows the nominal *P* value and NES for hematopoiesis. Heat map showing the gene expression changes of the top 30 genes after irradiation. Hematopoiesis genes are down-regulated after irradiation when compared to unirradiated control. SD from at least three treatments.

To identify molecular pathways and evaluate specific gene expression changes, we used Gene Set Enrichment Analysis (GSEA). GSEA analysis of three treatment groups (sham, day1, and day 10 following irradiation) was performed as described previously [13]. Differential gene expression profiles were generated for thousands of genes using RNA-seq data from sham (*n* = 3) versus day1 (*n* = 3) and day 10 (*n* = 3). Gene sets with member genes enriched in sham were discovered. Statistically significant gene sets were considered with a *P*-value < 0.05 and FDR (false discovery ratio) of < 0.25. The enrichment score (ES) reflects the degree to which a gene set is over-represented in the data set. The normalized enrichment score (NES) is the ES normalized for gene set size. The normalized enrichment score (NES) for the hematopoiesis genes was 1.7715 with a nominal *p*-value < 0.0001 (Figure 6). The hematopoiesis genes Hoxa9, Hoxa4, FLT3, HSPALL, and BCL2, were downregulated. The data suggest that hematopoiesis genes are downregulated in bone marrow in response to irradiation of thorax.

## DISCUSSION

Immunotherapy utilizes a patient’s own immune system to treat cancer and is at the forefront of cancer therapy. The CD4 and CD8 effector cells that identify and eliminate cancer cells play an essential role in cancer immunotherapy [5]. Monoclonal antibodies that target PD-1 or PD-L1 often referred to as checkpoint inhibitors are used to treat various cancers, including melanoma [14], non-small cell lung cancer [15], kidney cancer, bladder cancer, head and neck cancers, and Hodgkin lymphoma.

Radiation therapy (RT), which is an integral part of cancer management, causes radiation-induced lymphopenia (RIL). RIL is a significant clinical problem affecting treatment outcome and survival of cancer patients [2–4]. Persistence of RIL is associated with poor outcome for several carcinomas, including lung, colon, pancreas breast, sarcomas, and glioblastoma [2, 16].

The proposed mechanisms of RIL include secretion of galectin-1 from tumors but not from the host microenvironment or healthy tissues [6]. Studies with our mouse model showed RIL is independent of the site of irradiation, which is in agreement with the clinical data [3, 4, 8]. Unlike previous studies focusing on the direct effect of radiation on T-cells [5, 6]; we studied indirect effects on hematopoietic stem cells.

In this study, we used an innovative discovery platform and characterized the indirect effect of radiation on HSC. Within the BM, we identified the changes at the molecular level and cellular level. We found that the irradiated cells traffic to the bone marrow and deplete HSC and progenitor cells. These results support the model that RIL results from the indirect effect of radiation on the lymphocytes and stem cells in the bone marrow in addition to the direct effect on circulating lymphocytes.

We further evaluated thymus and spleen to gain a better understanding how secondary lymphoid organs respond to radiation. We found a significant reduction in the T-cells and B-cells in secondary lymphoid organs such as spleen and thymus. Our analysis revealed that both primary and secondary lymphoid organs are affected by radiation further supporting the idea that RIL results from the indirect effect of radiation on lymphoid organs.

Previously we found that lymphopenia is mainly caused by depletion of hematopoietic stem cells in the bone marrow [8]. In this study we further analyzed the bone marrow cells using CyTOF. Using CyTOF we found a reduction in CD11a expression in hematopoietic stem cells after irradiation when compared to sham irradiation. CD11a plays a significant role in the migration of lymphocytes and T cell development and is critical for the generation of common lymphoid progenitors (CLPs) [17]. The reduced expression of CD11a due to the indirect radiation could be (i) mobilizing the CD11a cells from the bone marrow into circulation (ii) preventing the development of lymphoid progenitor cells in the bone marrow, (iii) preventing mobilization of pre-T cells to the thymus, and (iv) preventing T-cell development in the Thymus. We found mobilization of CD11a cells from the bone marrow into circulation.

To identify the transcriptional changes of hematopoietic stem cells after irradiation, we utilized high-throughput RNA sequencing (RNA-Seq). We identified and quantitated gene expression changes (transcriptome changes) after irradiation. We used Gene set enrichment analysis (GSEA) to identify classes of genes that are over-represented in a broad set of associated genes. GSEA uses statistical approaches to identify significantly enriched or depleted groups of genes (http://software.broadinstitute.org/gsea/index.jsp). We found that hematopoiesis genes were down-regulated after radiation. The CyTOF analyses and bulk RNA-seq profiling indicated that pathways involved in immune regulation are drastically impacted after irradiation. This study identified potential candidates like CD11a and hematopoiesis genes like Hoxa9, Hoxa4, FLT3, HSPALL, and BCL2. These genes could play a potential role in iatrogenic immunosuppression. Future studies with defined genetic models will be required to answer these questions comprehensively.

## MATERIALS AND METHODS

### Reagents and antibodies

All antibodies for flow cytometry were from BD Biosciences (USA). APC-Cy7-CD3, FITC-CD4, PerCP-Cy5.5-CD8, and V450-CD19 antibodies were used to stain blood. BM mononuclear cells were stained with PerCP-Cy5.5 conjugated lineage markers (Mac-1, Gr-1, B220, CD3, and Ter119), PE-Sca-1, APC-Cy7-c-kit, APC-Flt3, and FITC-CD34.

### Irradiations

All studies were performed in accordance with the guidelines of the Institutional Animal Care and Use Committee and with protocols approved by the Washington University Division of Comparative Medicine. Six to eight week old female C57BL/6 mice were obtained from Charles River. Mice were anesthetized with 2% isoflurane prior to irradiations to the thorax or the head (Supplementary Figure 1). The mice were irradiated with 1.8 Gy/day for five consecutive days. In some experiments, 200 μl of blood was drawn from mice, irradiated with one dose of 6 Gy *ex vivo*, and re-injected autologously to the respective mice. Mice and blood were irradiated using RS-2000 (Rad Source) irradiator at a dose rate of 1 Gy/min with 160 kVp X-rays.

### Flow cytometry

The peripheral blood cells and splenocyte cells from various treatments were stained with anti- CD16/32, CD3, CD4, CD8, and CD19 antibodies. For thymocytic cells CD117, CD44, CD25, CD127, CD3, CD4 and CD8 were used to stain different population. Hematopoietic stem and progenitor cells were stained with lineage markers as described earlier [18]. We used following antibodies: CD117, Ly-6A/E, CD34, CD135, CD127, CD48, CD150 and CD16/32 from BD biosciences and Mac-1, Gr-1, CD4, CD8, B220, CD3, and Ter119 from Biolegend. Cells were analyzed by MACSQuant Analyzer flow cytometer (Miltenyi Biotec) and data analyzed with FlowJo software (Tree Star Inc.).

### DiD staining

We isolated peripheral blood cells (PBCs) from 200 μl of mouse blood, resuspended the PBCs in autologous plasma and irradiated them *ex-vivo*. The irradiated PBCs in plasma were incubated overnight at 37ºC. For tracking experiments, cells were labeled with a fluorescent membrane dye, Vybrant DiD (ThermoFisher Scientific) according to the manufacturer’s instructions.

### Mass cytometry (CyTOF)

Metal-tagged antibodies were purchased from Fluidigm or custom-conjugated using the Maxpar ×8 Antibody Labeling Kit as per manufacturer’s instructions (Fluidigm). All custom-conjugated antibodies were titrated. For staining, 3 × 10^6^ bone marrow cells were stained with surface antibodies (Supplementary Table 1) for 1 h at 4°C in CyFACS buffer (0.1% BSA, 0.02% NaN2, 2 mM EDTA in CyPBS, Rockland). Cells were stained for viability with 2.5 μM cisplatin (Enzo life sciences) according to a standard protocol [19, 20]. Cells were washed three times and stained with Cell-ID intercalator according to the manufacturer’s instructions (Fluidigm).

Data were analyzed using viSNE (Cytobank), with uniform sampling, 10% down-sampling, and clustered on the parameters shown in Supplementary Table 1. Using the density plots, various populations were gated in the tSNE1/2 fields. The viSNE-gated populations were then assessed for the median expression of indicated markers, as well as percent positive for the indicated markers.

### RNA-Seq

Mice were irradiated with 5 doses of 1.8 Gy to the thorax, and sham-irradiated mice served as controls. The bone marrow cells were harvested from femurs by flushing them with 1 ml of PBS at days 1 and 10 following IR. The mRNA was isolated from bone marrow cells using the miRVANA kit (Ambion). RNA sequencing was performed by Genome Technology Access Center (GATC), Washington University in St. Louis. Gene counts were derived from the number of uniquely aligned unambiguous reads by Subread: feature Count version 1.4.5. Transcript counts were produced by Sailfish version 0.6.3. Sequencing performance was analyzed for a total number of aligned reads, the total number of uniquely aligned reads, genes, and transcripts detected, ribosomal fraction known junction saturation and read distribution over known gene models with RSeQC version 2.3.

Genes or transcripts not expressed in any sample or less than one count-per-million in the minimum group size minus one were excluded from further analysis. Performance of the samples was assessed with a Spearman correlation matrix and multi-dimensional scaling plots. Gene/transcript performance was evaluated with plots of residual standard deviation of every gene to their average log-count with a robustly fitted trend line of the residuals. Differentially expressed genes and transcripts were then filtered for FDR adjusted *p*-values less than or equal to 0.05.

### Gene Set Enrichment Analysis (GSEA)

GSEA analysis of 3 groups (sham, day1 and day 10 following irradiation) was performed as described previously [13]. Briefly, differential gene expression profiles were generated using RNA-seq data for sham (*n* = 3), day1 (*n* = 3) and day 10 (*n* = 3). Statistically significant gene sets were considered with a *P* value < 0.05 and FDR (false discovery ratio) < 0.25. The enrichment score (ES) reflects the degree to which a gene set is over-represented in the dataset. The normalized enrichment score (NES) is the ES normalized for gene set size.

### Statistical analyses

For all the studies, at least 3–4 mice were used for each treatment. All pair-wise comparisons between treatment groups were performed using moderated *t*-test. A *p*-value of < 0.05 was considered statistically significant. These analyses were performed in Prism 7 (GraphPad Software, La Jolla, CA, USA), and statistical significance was indicated in each graph where appropriate.

## SUPPLEMENTARY MATERIALS


