# Supplementary material for: Radiation induces iatrogenic immunosuppression by indirectly affecting hematopoiesis in bone marrow
Source: Oncotarget. 2020 May 12;11(19):1681–90. doi: 10.18632/oncotarget.27564 (PMC7233802; doi:10.18632/oncotarget.27564)
Supplement: Supplementary file 1 [file oncotarget-11-1681-s001.pdf]

Radiation induces iatrogenic immunosuppression by indirectly affecting hematopoiesis in bone marrow

SUPPLEMENTARY MATERIALS

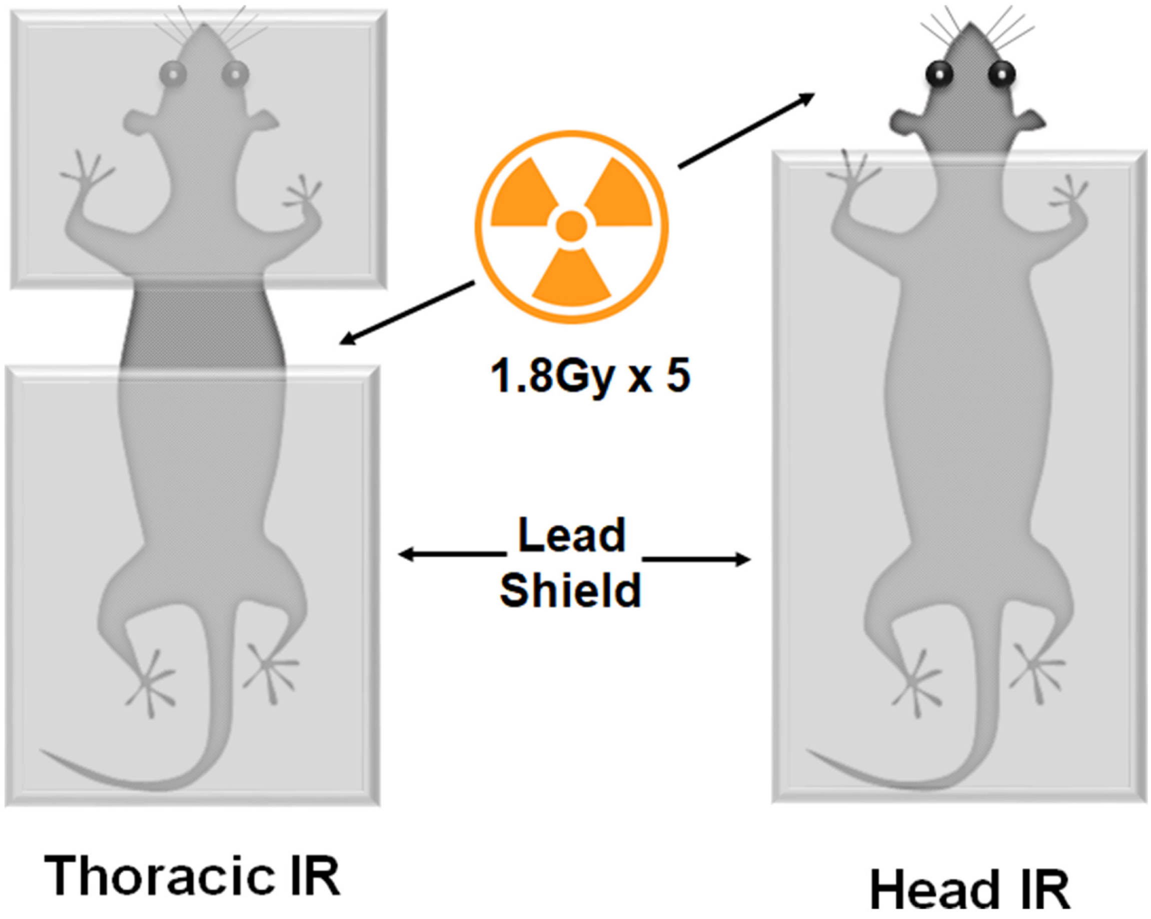

Supplementary Figure 1: Mice were anesthetized with 2% isoflurane prior to irradiations to the thorax or the head. The mice were shielded with lead to expose the head or thorax and protect the other organs from radiation as shown in the figure. The mice were irradiated with 1.8 Gy/day for five consecutive days.

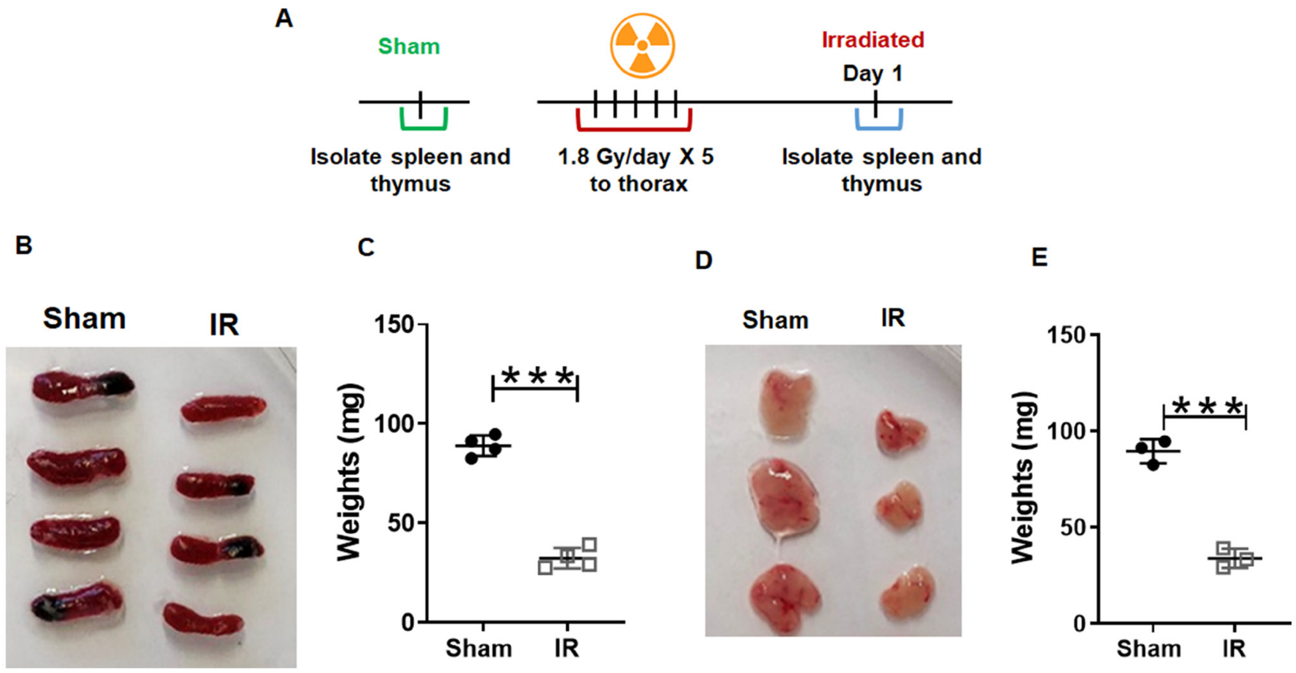

Supplementary Figure 2: Radiation depletes spleen and thymus. (**A**) Schematic representation of the treatment plan for mice. The mouse thorax were irradiated (1.8 Gy ×5) spleen and thymus were harvested 1 day after irradiation and untreated mice were used as controls. Shown are the pictures (**B** and **D**) and weights (**C** and **E**) of the spleen and thymus. SD from at least three treatments.

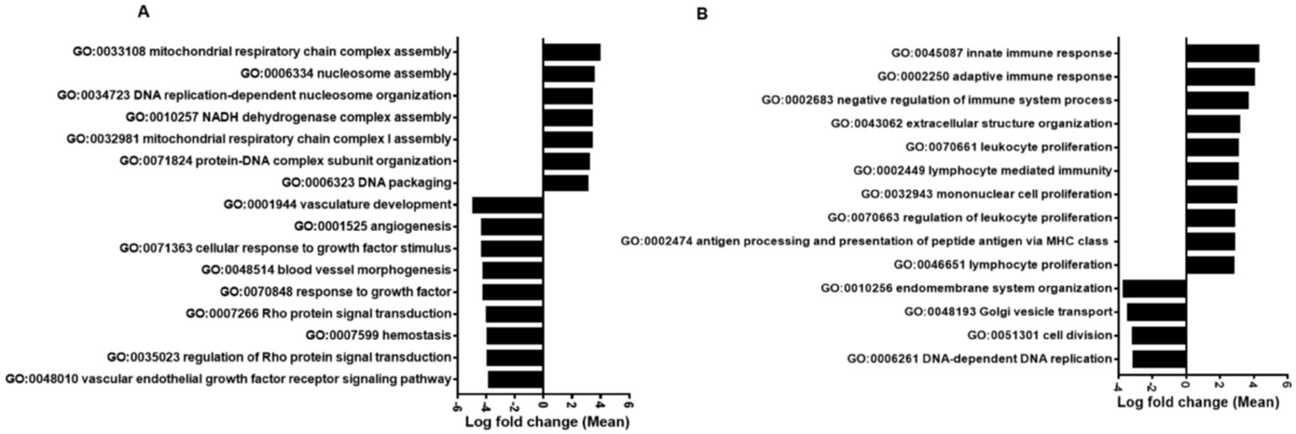

Supplementary Figure 3: Mice were irradiated with 5 fractions of 1.8 Gy in the thoracic region and the bone marrow cells were collected form the femur at day 1 or day 10. Sham mice were used as controls. Shown are the fold change of the GO biological processes. Bar graph showing the mean log fold changes of the GO biological processes at day 10 compared to sham mice (**A**) and Day 10 compared to day 1 (**B**). SD from at least three treatments.

Supplementary Table 1: Mass cytometry panel design

Label	Target	Clone
089Y	CD45	30-F11
141Pr	Ly-6G/C (Gr-1)	RB6-8C5
143Nd	CD41	MWreg30
144Nd	CD16/32	93
145Nd	CD11a	M17/4
146Nd	CD71	C2 (C2F2)
148Nd	CD11b (Mac-1)	M1/70
150Nd	CD44	IM7
153Eu	CD8a	53-6.7
154Sm	CD48	HM48-1
156Gd	CD90.2	30-H12
159Tb	CD45R/B220	RA3-6B2
160Gd	CD62L	MEL-14
162Dy	TER-119	TER-119
164Dy	Flt3	
165Ho	CD3e	145-2C11
167Er	CD150	TC15-12F12.2
169Tm	Ly-6A/E (Sca-1)	D7
170Er	CD161 (NK1.1)	PK136
172Yb	CD4	RM4-5
173Yb	CD117 (ckit)	2B8
174Yb	CD229	Ly9ab3
175Lu	CD127 (IL-7Ra)	A7R34
176Yb	CD61-APC	
209Bi	CD11c	N418

The metal isotope tag, target name and antibody clone is shown for a panel designed to phenotype mouse bone marrow cells.
